# Sudden Death Induced by Acute Inhalation of Aerosolized Carfentanil

**DOI:** 10.26502/acbr.50170440

**Published:** 2025-03-19

**Authors:** Xiuping Gao, Jianguo Zhuang, Zikuan Chen, Shan Shi, Fadi Xu

**Affiliations:** Department of Physiology, Lovelace Biomedical Research Institute, Albuquerque, New Mexico, USA

**Keywords:** Depressed ventilation, Hypotension, Bradycardia, Apnea, Ataxic breathing

## Abstract

Bilateral Inhalation of carfentanil (CRF) aerosol could rapidly induce death in humans and CRF aerosol exposure induces respiratory depression in animals; however, the dynamic progression of cardiorespiratory failure leading to sudden death induced by acute CRF aerosol exposure remains unclear. This study aimed to establish a rat model that allows dynamically characterizing the cardiorespiratory failure prior to death following acute exposure to a lethal concentration of CRF aerosol. Two groups of anesthetized and spontaneously breathing rats were exposed to aerosolized vehicle and CRF (4 mg/m^3^) for 10 minutes respectively. CRF exposure resulted in 100% mortality among the tested rats. The cardiorespiratory responses were characterized sequentially by: immediate ventilatory depression resulting from rapidly developed bradypnea, persistent ventilatory depression, and hypotension and significant irregularities in both breathing (ataxic breathing) and heart beat rhythms that ultimately caused ventilatory and cardiac arrest at 7.7 ± 0.6 and 9.0 ± 0.7 minutes, respectively, after the onset of CRF exposure. Our results establish a rat model of cardiorespiratory failure and sudden death resulted from acute exposure to CRF aerosol. This model may facilitate further investigation into the mechanisms underlying cardiorespiratory failure and the development of potential countermeasures.

## New & Noteworthy

Inhalation of carfentanil (CRF) aerosol rapidly causes deaths in humans, but the development of cardiorespiratory failure prior to death is unknown. We established a rat model of CRF exposure-induced sudden death occurring within several minutes. CRF exposure causes bradypnea and ataxic breathing associated with bradycardic and hypotension, resulting in death.

## 1. Introduction

The use of potent synthetic opioids induces more than 500,000 deaths in the United States over the last 20 years. It has become a public health concern globally due to their fatal intoxications, in which carfentanil (CRF) is the second most frequently used after fentanyl [1]. CRF is the most dangerous fentanyl derivative, contributing to life-threatening hospital admissions and fatalities in the North American [2–5]. For example, a report indicated that among 11,045 opioid overdose deaths occurring in 10 states of the United States during the period of July 2016–June 2017, 1236 (11.2%) decedents tested positive for carfentanil [6]. The fatality of CRF administered via injection, insufflation and inhalation is emanated from a rapid and severe respiratory depression, which could lead to apnea, respiratory arrest, and death in overdose cases [2]. CRF has several unique features making it especially dangerous, such as a potency 10,000 times that of morphine and 100 times that of fentanyl [7], the availability online as a ‘research chemical’ or ‘pharmaceutical intermediate’ and especially, the low molecular weight and high lipophilicity [3]. Because of the highly absorbable ability through inhalation, CRF was infamously used as a “poison” gas to incapacitate Chechen rebels in the 2002 Moscow theater incident, where aerosolized CRF was introduced into the ventilation system, resulting in the deaths of 125 hostages [8,9]. In fact, exposure to CRF aerosol through inhalation, an noninvasive approach, evidently poses extra potential risks for illicit users (https://www.dea.gov/press-releases/2016/09/22/dea-issues-carfentanil-warning-police-and-public).

While the impacts of opioids on respiratory depression have been extensively investigated, there is still an urgent need to characterize the progression of cardiorespiratory failure prior to death induced by exposure to acute CRF aerosol owing to the unique features of CRF mentioned above.

Previous studies in animals have shown that aerosolized CRF at 0.4 and 4 mg/m^3^ induces dyspnea/labored breathing and ~35–55% respiratory depression associated with hypotension and bradycardia at the end of the 15 min exposure in mice [10,11]. Similarly, CRF exposure at 0.72 mg/m^3^ for 20 min results in apneas and respiratory depression in ferrets, leading to death in one of tested ferrets [12]. Instead of aerosol exposure, systemic injection of CRF also causes remarkable ventilatory depression in rats and mice [13–18]. However, so far as we know, none of these studies has been focused on revealing the development of cardiorespiratory failure prior to death resulted from acute exposure to CRF, which has hindered the relevant mechanistic study in-depth. Thus, the goal of this study was to dynamically characterize the cardiorespiratory failure leading up to death, and thereby to establish a rat model of sudden death induced by acute exposure to an overdose of aerosolized CRF.

To achieve the goal, our experiments were performed in two groups of anesthetized and spontaneously breathing rats exposed to aerosolized vehicle and an overdose of CRF at 4 mg/m^3^ for 10 minutes, respectively. The cardiorespiratory responses were compared between the two groups.

## 2. Materials and Methods

### Animal use.

Seventeen adult male pathogen-free Sprague-Dawley rats (250–350 g) were purchased from Charles River Laboratories, Inc. (Wilmington, MA). All rats were housed in filter top cages in the animal facility of Lovelace Biomedical Research Institute with 12:12 h light/dark cycle and had free access to food and water ad libitum. The rooms were constantly ventilated under conditions of controlled temperature (24–25°C) and humidity (45% ± 5%). The animals were quarantined for one week before experiments. Experiments were performed during 9:00 and 17:00 hours to avoid influence of the circadian rhythm. The experimental protocols (FY23–010) were conducted in accordance with the Guide for the Care and Use of Laboratory Animals and approved by the Institutional Animal Care and Use Committee (IACUC), which is accredited by the Association for Assessment and Accreditation of Laboratory Animal Care International, USA. Experiments were designed to minimize the number of animals included in the study.

### Chemicals.

Urethane (item # U2500) and methanol (item # 34860–100ML-R) were purchased from Sigma-Aldrich (St. Louis, MO). Carfentanil (CRF) was purchased from Cayman Chemical (item # 31340 in the form of 1 mg/mL solution in methanol) and diluted with normal saline to a concentration of 20 μg/mL for aerosol generation.

### Animal preparation and aerosol inhalation.

**Figure 1:**
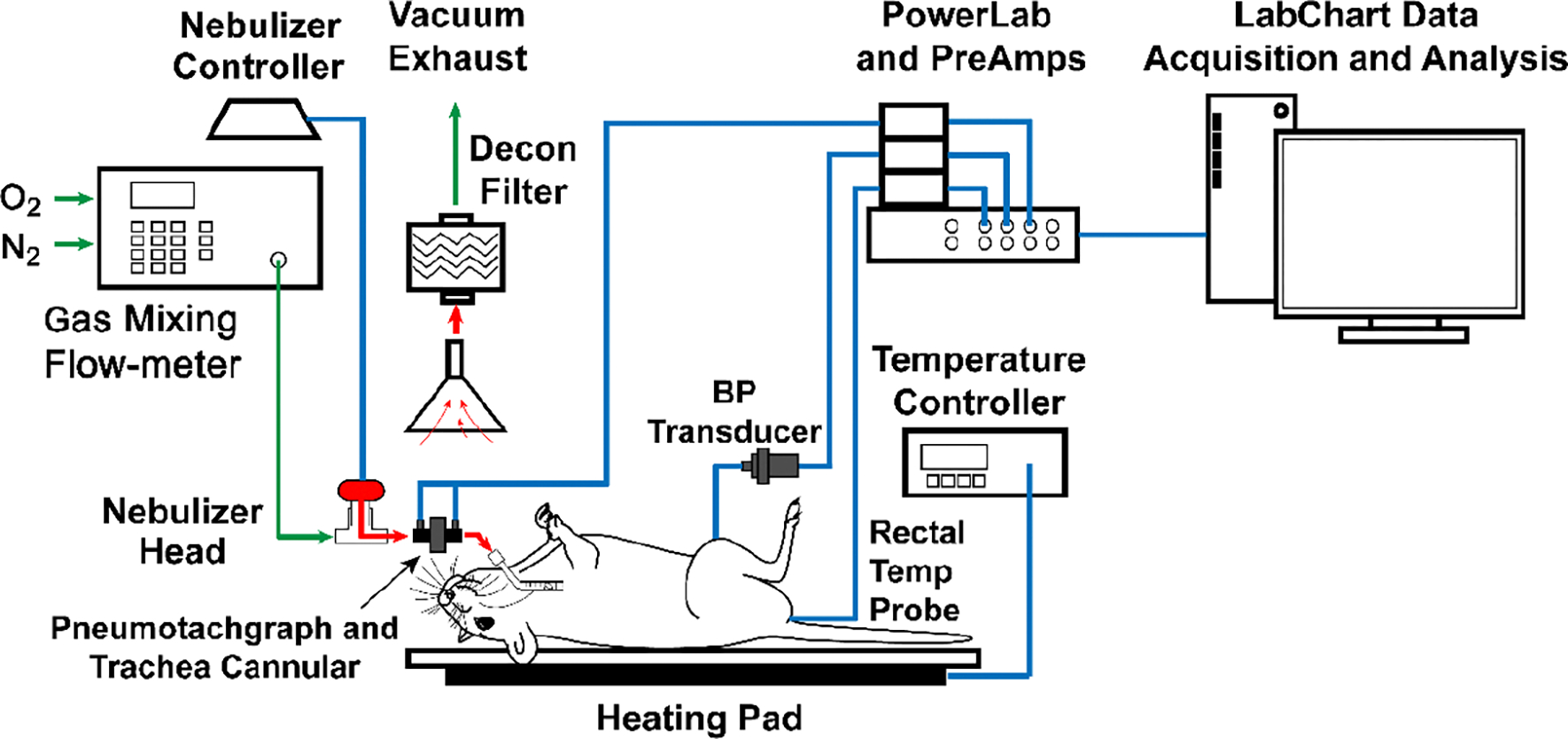
Cartoon showing the animal preparation and the configuration of aerosolized CRF inhalation exposure system.

The animals were anesthetized with urethane (1.2 g/kg, ip), with supplemental doses (0.3 g/kg, ip) administered as needed to completely suppress eye-blink and limb- withdrawal reflexes throughout the experiment. As illustrated in Figure 1, the anesthetized and spontaneously breathing rat was placed in a supine position with the trachea transected and cannulated [19]. The tracheal cannula was connected to a pneumotachograph (item # MLT1L, ADInstruments Inc., Colorado Springs, CO) to record minute ventilation (VE), tidal volume (VT), respiratory frequency (fR), inspiratory and expiratory duration (TI and TE), and duty cycle (TI/TTot). Additionally, the right femoral artery was cannulated to monitor the arterial blood pressure (ABP) and heart rate (HR). The animal’s core temperature was monitored using a rectal probe and maintained at 36.5–37.0°C with a heating pad and radiant heat lamp throughout the experiment.

Gases (30% O2 in nitrogen) were mixed by using a gas mixing flow meter (GF-3MP, Cameron Instrument Co., Port Aransas, TX) with an output flow rate of 1.0 L/min. The gas mixture was delivered to a tube (diameter 2.0 cm), while a vibrating mesh nebulizer (normal saline output 0.33 ml/min; Ireland Ltd., Galway Ireland, AG-AL1000) was positioned in the middle of the tube via a T-type connection. The aerosol ejected by the nebulizer was mixed with the airflow in the tube that was loosely jacketed to the pneumotachograph connected to the tracheal cannula for delivery to the animal. The aerosol exposure duration was 10 minutes, with an effective nebulizer output of 0.20 ml/min for both vehicle and CRF solutions. The aerosol had a volume median diameter of 2.5–4.0 μm, as indicated by the manufacturer. A funnel positioned over the pneumotachograph was connected to the vacuum with inline filters (Pall BB50T Breathing Circuit Filters, Pall Corp, NY) to filter the exhausted aerosol for decontamination. The aerosol exposure setup was positioned in a standard exhaust fume hood (size: 3 × 6 ft).

### Experimental protocols.

The goal of this study was to establish a rat model of sudden death induced by acute exposure to an overdose of aerosolized CRF. A pilot study was conducted to determine a lethal concentration of CRF aerosol. The anesthetized and spontaneously breathing rats were exposed to aerosolized CRF at low (0.8 mg/m^3^), medium (2 mg/m^3^) and high (4 mg/m^3^) concentration for 10 min in three groups (n = 2/group). These CFR aerosol concentrations are comparable to those previously used in mice [10]. The animals’ breathing was observed for up to one hour following the onset of the exposure.

Because only exposure to CRF at high concentration resulted in death, the following experiments were performed in the anesthetized and spontaneously breathing rats that were randomly divided into two groups: one group received vehicle aerosol (Veh, 2% V/V methanol in normal saline; n = 5) and the other group received CRF (20 μg/mL in vehicle) at the high concentration (4 mg/m^3^, n = 6). The exposure lasted for 10 minutes. At least five minutes after stabilization, ventilation (VE, VT and fR), respiratory timing (TI, TE and duty cycle), as well as ABP and HR were simultaneously and continuously recorded before exposure and during aerosol exposure.

### Data Acquisition and Statistical Analysis.

Raw data were digitized and recorded using a PowerLab/8sp unit (model ML 785, ADInstruments Inc., Colorado Springs, CO) and a computer with LabChart Pro 7 software. Their derived data included VE, VT, fR, HR, and mean ABP (MABP). Additionally, the variation of respiratory intervals is a more sensitive index than fR for reflecting changes of respiratory control [20]. We applied the Poincaré analysis over different time frames using consecutive respiratory interval values from respiratory flow and heart beat interval data derived from ABP pulses in each animal, plotting the previous interval against the next, as previously reported [21,22]. The standard deviations were calculated perpendicularly to (SD1) and along the line of identity (SD2). In the rats exposed to CRF, cardiorespiratory data were collected before aerosol exposure (baseline) and at 0.5, 1, 1.5, and 2 minutes after the onset of the exposure during the initial period of the exposure. Because the timing of ventilatory arrest was varied among individuals, the period from 2 minutes to the last breath was evenly divided into four segments, with cardiorespiratory data collected at the end of each segment (3.4 ± 0.2, 4.8 ± 0.3, 6.1 ± 0.4, and 7.6 ± 0.6 minutes, respectively). Two additional measurements of MABP and HR were collected after ventilatory arrest at 8.1 ± 0.6 and 8.9 ± 0.7 min to reflect the rebound ABP response (at the peak and the following lowest ABP levels) just before cardiac arrest. Respiratory and heart rate interval SD1 and SD2 values were calculated from the data segments between the aforementioned time points. Data from animals exposed to vehicle aerosol were collected at the same averaged time points as listed in the CRF group. The evoked cardiorespiratory responses were represented as the Δ% changes of the corresponding baseline values, while their baseline values and the data on the respiratory interval and HR variability (SD1 and SD2) were represented as absolute values. All data were reported as the mean and standard error (mean ± SE). Group Student’s t-test was used to compare the baselines between the two groups. Two-way ANOVA with repeated measures was used to analyze the evoked responses at different time points in each group and between the rats exposed to vehicle and CRF. Multiple comparison results were corrected using FDR-BH method, with P-values < 0.05 considered significant.

## 3. Results

### Ventilatory responses to the aerosolized CRF consist of three phases.

**Figure 2:**
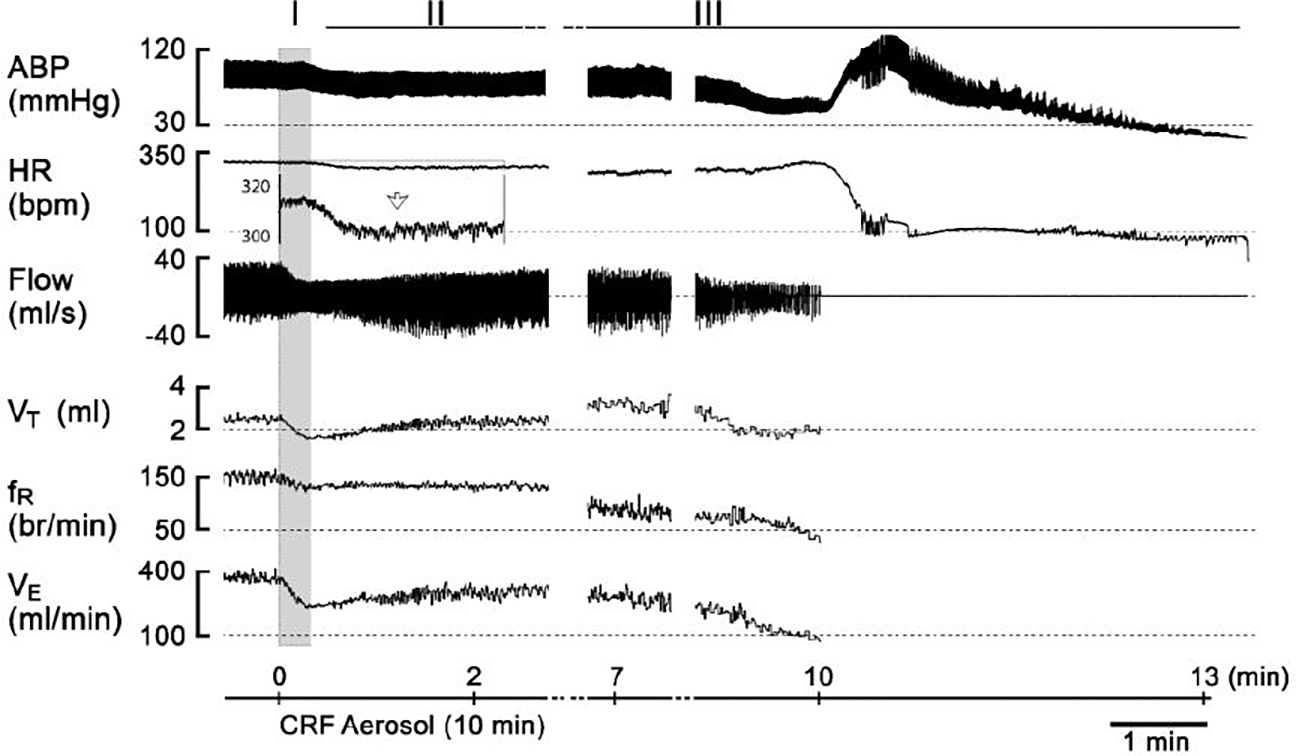
Typical recordings to show the three phases of cardiorespiratory response to aerosolized CRF for 10 min in an anesthetized and spontaneously breathing male rat. The inset presents the enlargement of the signals of heart rate. The traces from the top to bottom are arterial blood pressure (ABP), heart rate (HR), airflow, tidal volume (V_T_), respiratory frequency (f_R_), and ventilation (V_E_). The CRF exposure induces three phases of cardiorespiratory responses (I – III).

**Figure 3:**
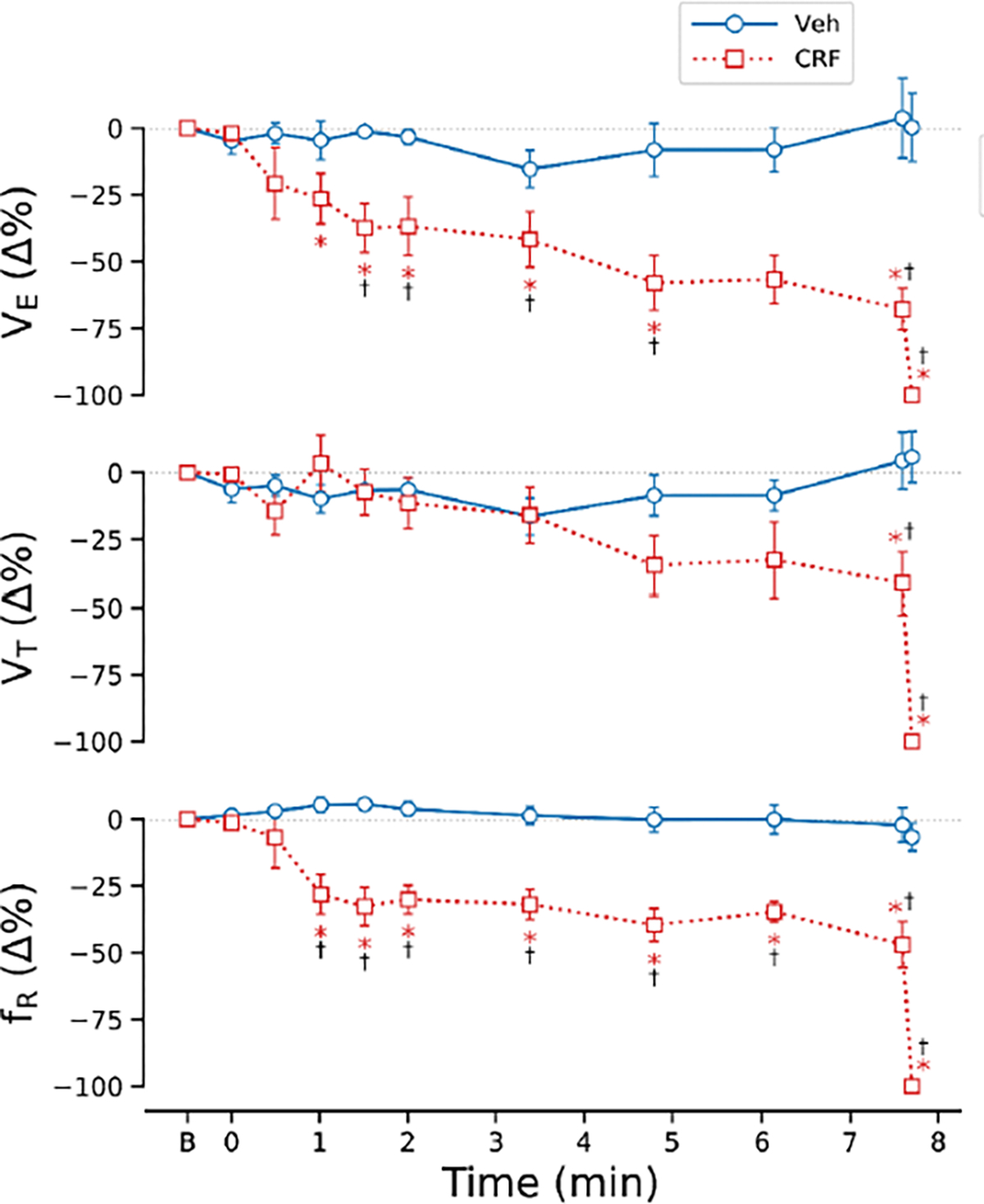
A: Comparison of the respiratory response (VE, VT and fR) to aerosolized vehicle (Veh) and carfentanil at 4 mg/m3 (CRF) for 10 min in anesthetized and spontaneously breathing rats. n = 5 and 6 for Veh and CRF. * P < 0.05, compared to corresponding baseline “0” values; † P < 0.05, compared to the corresponding Veh. “B” on x-axis represents baseline.

Figure 2 exhibits the typical ventilatory response to CRF in an anesthetized and spontaneously breathing rat. The responses consist of three phases: the immediate hypoventilation, the persistent hypoventilation, and the exacerbation of hypoventilation that ultimately led to cardiorespiratory failure. The group data corroborated these findings (Figure 3). Veh exposure did not significantly change the breathing. In contrast, CRF exposure resulted in 100% mortality (8/8 rats). The cardiorespiratory responses to CRF were characterized by a sequential progression through the three phases mentioned above. At phase III, VE, VT, and fR decreased markedly and rapidly, leading to ventilatory arrest occurring at 7.7 ± 0.6 minutes after the onset of CRF exposure. It should be noted that there was no significant difference in the baseline cardiorespiratory variables between the two groups of the rats (Table 1).

### CRF affects respiratory timing.

**Figure 4:**
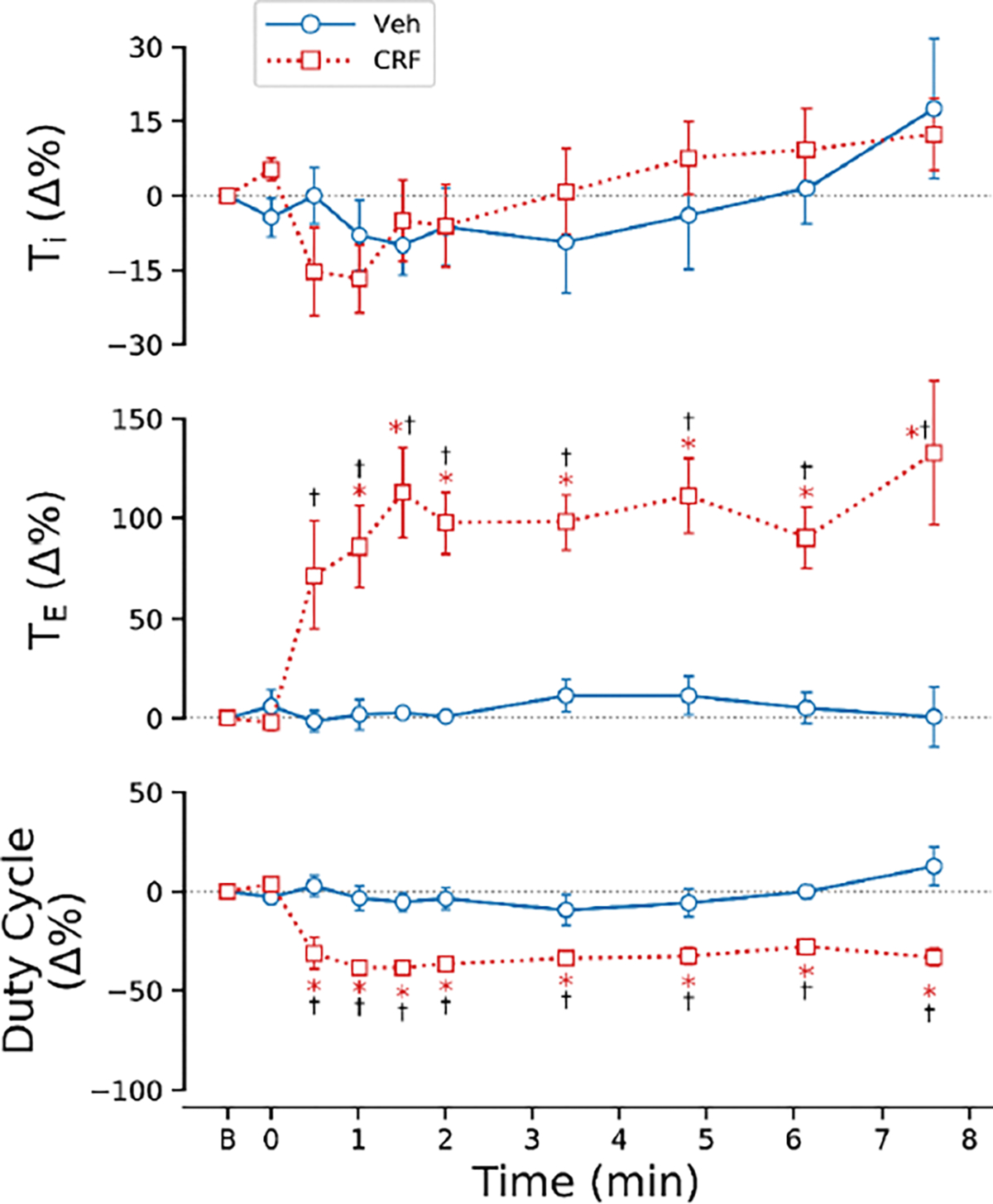
Comparison of respiratory timing (TE, TI and TI/TT) in response to aerosolized vehicle (Veh) and carfentanil at 4 mg/m3 (CRF) for 10 min in anesthetized and spontaneously breathing rats. n = 5 and 6 for Veh and CRF. * P < 0.05, compared to corresponding baseline “0” values; † P < 0.05, compared to the corresponding Veh. “B” on x-axis represents baseline.

As illustrated in Figure 4, Veh exposure failed to alter the respiratory timing, whereas CRF exposure resulted in significant changes. Specifically, CRF exposure markedly increased TE and decreased the duty cycle (TI/TT). A rapid prolongation of TE to ~100% and a reduction of the duty cycle to ~35% were observed within one minute after the onset of CRF exposure, and these changes were maintained throughout the exposure until the ventilatory arrest occurred. TI remained unchanged with both Veh and CRF exposure, and there was no significant difference in TI between the rats exposed to Veh and those exposed to CRF.

### Ataxic breathing occurs prior to death.

**Figure 5:**
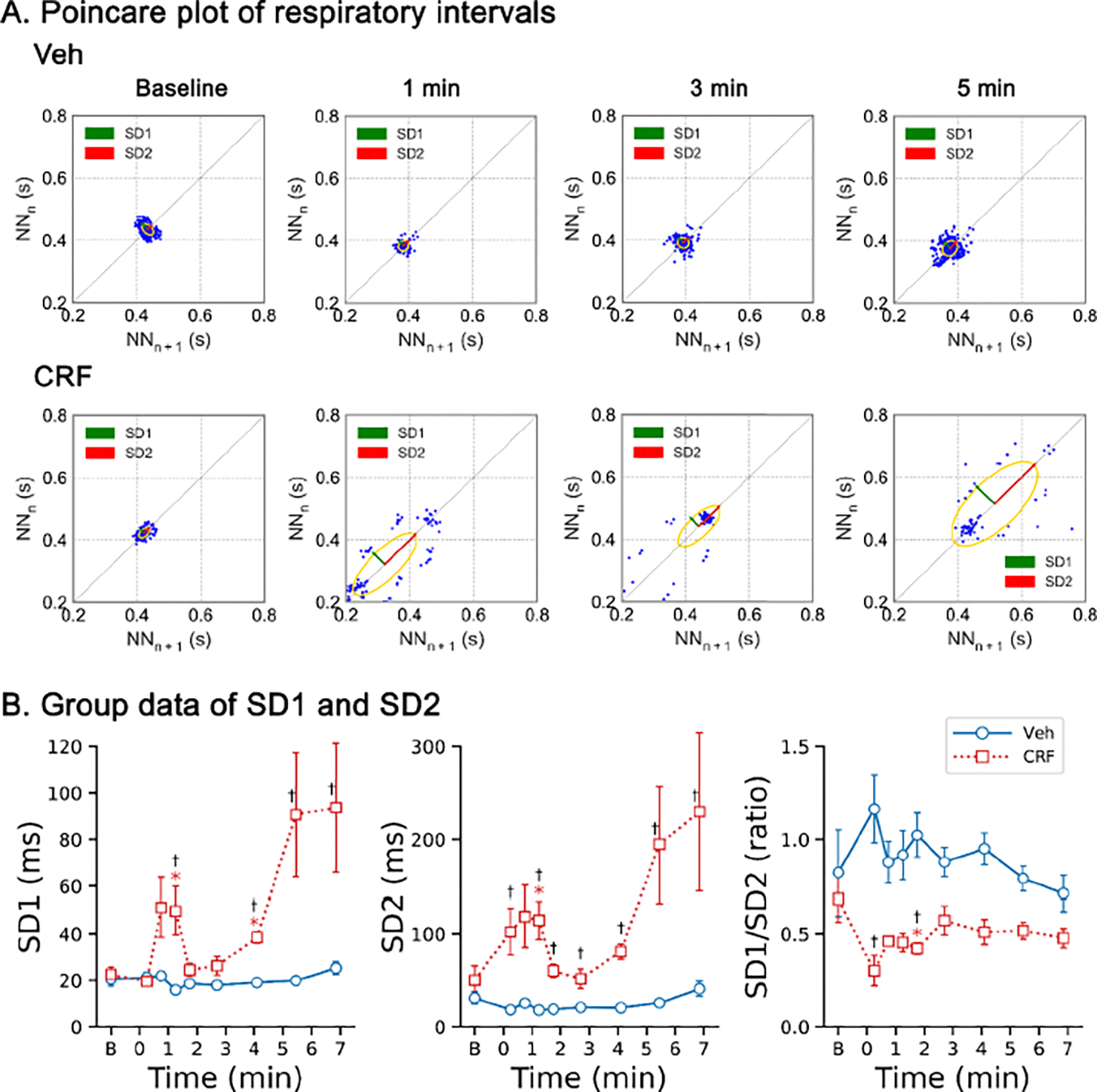
A: Typical Poincaré plots illustrating the variation of the respiratory intervals (NNn vs. NNn+1) in two rats exposed to vehicle (Veh) and carfentanil (CRF), respectively. The ellipse area describes the distribution of the points with the width of the standard deviation perpendicular to (SD1) and along the line of identity (SD2). B: the corresponding group data for SD1 and SD2. n = 5 and 6 for Veh and CRF. * P < 0.05, compared to corresponding baseline “B” values; † P < 0.05, compared to the corresponding Veh. “B” on x-axis represents baseline.

We compared the variability of the respiratory intervals between the rats exposed to Veh and CRF. As compared to the rats exposed to Veh, the rats exposed to CRF developed ataxic breathing which caused significant increases in both SD1 and SD2, with an initial peak occurring approximately one minute after the onset of CRF exposure (Figure 5). Following a temporary recovery that lasted about 2 minutes, SD1 and SD2 values were elevated again, and the values remained high until ventilatory arrest occurred due to CRF exposure. In contrast, Veh exposure did not affect the variability of the respiratory intervals.

### CRF exposure suppresses cardiovascular activities.

**Figure 6:**
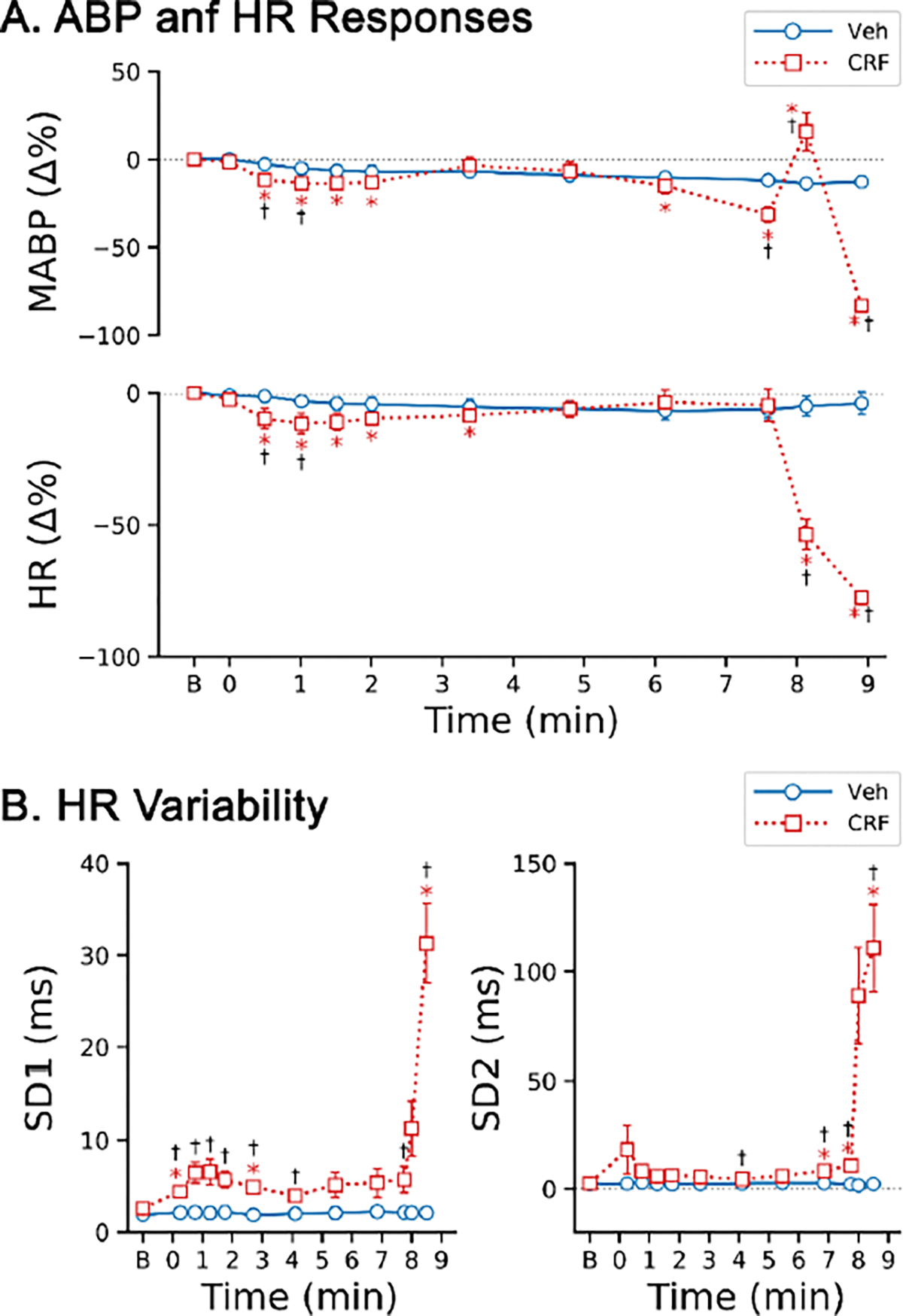
The responses of mean arterial blood pressure (MABP) and heart rate (HR) to inhalation of aerosolized vehicle (Veh) and carfentanil (CRF) at 4 mg/m^3^ for 10 min in anesthetized and spontaneously breathing rats. n = 5 and 6 for Veh and CRF. * P < 0.05, compared to corresponding baseline “0” values; † P < 0.05, compared to the corresponding Veh. “B” on x-axis represents baseline.

Figure 6 exhibits the impact of CRF on cardiovascular activities. Group data indicated that exposure to CRF aerosol for 10 minutes caused a slight but significant hypotension within the first minute, followed by a recovery of MABP which lasted for ~5 min. Thereafter, MABP continued to decline, with a more rapid decrease occurring in the later stages for rats exposed to CRF. A few seconds after ventilatory arrest, MABP was quickly raised to a short peak within about 15 seconds before steadily declining to a level near 0. With respect to HR, a temporary bradycardia was observed 1–2 min after the onset of CRF exposure followed by a recovery that lasted until the occurrence of ventilatory arrest. After ventilatory arrest, HR dropped rapidly, leading to a subsequent cardiac arrest occurring at 9.0 ± 0.7 min. HR variability analysis revealed an early increase in SD1 in the rats exposed to CRF, corresponding to the bradycardic response temporally. A later SD1 increase, along with an elevation in SD2, was noted after ventilatory arrest during the quick decline in HR. Contrary to the rats exposed to CRF, Veh exposed rats did not exhibit significant changes in MABP and HR levels, or HR variability.

## 4. Discussion

Overdose CRF administered through injection, insufflation, and inhalation has been reported to cause a rapid and severe respiratory depression (apnea and respiratory arrest) in the North American [2–6]. Actually, inhalation of CRF has been linked to fatalities in humans in the 2002 Moscow theater incident [8,9]. To date, the dynamic development and characteristics of cardiorespiratory failure prior to death have not been determined. CRF produces typical opioid effects in animals, including antinociception, catalepsy, and respiratory depression. Exposure to CRF aerosol has been shown to induce respiratory depression (dyspnea/labored breathing) and bradycardia in mice and ferrets [10–12]. CRF administered through intravenous or subcutaneous injection (1 – 20 μg/kg) markedly depressed ventilation primarily by decreasing f_R_, and hypoxemia lasting for at least 1.5 hours in rats [13,14,16]. Intraperitoneal injection of CRF (1– 30 ug/kg) reduced VE by 20 – 80% in a dose-dependent manner in mice [15,17,18]. However, none of the above animal studies characterized the cardiorespiratory failure prior to CRF exposure-induced death. In the present study, we found that a 10-minute exposure to CRF (4 mg/m^3^) led to 100% mortality among anesthetized and spontaneously breathing rats (8/8). Three phases of the cardiorespiratory responses were observed in these rats: the initial hypoventilation; the persistent hypoventilation; and subsequently exacerbated hypoventilation (ataxic breathing), bradycardia (irregular HR) and hypotension that eventually caused ventilatory and cardiac arrest at 7.7 ± 0.6 and 9.0 ± 0.7 min after the onset of the exposure. Therefore, our results establish, for the first time, a rat model revealing the dynamic development of cardiorespiratory failure prior to CRF exposure-induced sudden death.

The evidence is accumulating to show both peripheral and central mechanisms involving the opioid- induced respiratory responses. Peripherally, IV bolus injection of a low dose of opioids (fentanyl) is able to trigger an immediate vagal-mediated brief apnea in anesthetized rats [23–26]. Similarly, IV bolus injection of 50 μg/kg of fentanyl produces an immediate sustained apnea (lasting for ~1.5 min) followed by respiratory depression in anesthetized rats, and the apnea, but not the subsequent respiratory depression, is abolished by vagal C-fiber degeneration [27]. Centrally, activation of opioid receptors, particularly mu-opioid receptors, in the brainstem is well known to be predominantly responsible for the opioid-induced respiratory depression [8,28–30]. The respiratory-related neurons within the preBötzinger complex, parabrachial nucleus/Kölliker-Fuse complex, retrotrapezoid and parafacial nucleus, raphe area and nucleus tractus solitarius in the brainstem are all pivotal in genesis of the opioid-induced respiratory depression [19,31–42]. In the present study, the significant respiratory depression was observed approximately one minute after the onset of CRF exposure, suggesting that this response is mediated primarily by central mechanisms. It is warranted to determine in the future whether CRF exposure induces respiratory depression (and subsequent death) through activation of central opioid receptors, particularly mu-opioid receptors, and to identify the specific brainstem regions responsible for the pathophysiological genesis of respiratory failure.

We examined the variation of the respiratory intervals and found that ataxic breathing occurred rapidly, within 1–2 minutes after the onset of CRF exposure, and reappeared just prior to death in the rats. Normal variability in breath intervals among mammals possesses an optimal ranging from an absence of any variability to a clearly periodic breathing pattern influenced by excitatory and inhibitory inputs from a variety of neural feedback loops at different states [43]. The normal breathing rhythm is thought to be generated by respiratory-related neurons within the medullary preBötzinger complex, particularly those neurons expressing neurokinin-1 receptor to function as respiratory pacemakers [44–46]. Dysfunction of these neurons leads to ataxic breathing in mice [22]. Indeed, activation of mu-opioid receptors in the preBötC region alters respiratory rhythm preferentially through inhibiting local neurons expressing neurokinin-1 receptor [47]. Bilateral microinjection of opioid mu-receptor agonist DAMGO into the preBotzinger Complex reduces peak phrenic amplitude and induces irregular (ataxic) patterns of breathing in vagotomized rabbits [48]. In agreement, chronic use of opioids is associated with a higher risk for ataxic breathing and central sleep apnea in clinical settings [49,50]. particularly In the present study, CRF significantly increased the variability of breath intervals, peaking initially during the 1^st^-2^nd^ minutes of CRF exposure and again prior to death (Figure 5). Our data indicate a possibility that CRF exposure may cause ataxic breathing by binding to opioid receptors, mu- opioid receptors, in the neurons expressing neurokinin-1 receptor in the preBötzinger complex. This finding aligns with previous reports indicating that the risk of opioid-induced death correlates more closely with progressive breathing irregularity than with severe reductions in respiratory rate [51,52].

In the present study, the ventilatory arrest usually occurs approximately eight minutes after the onset of CRF exposure. In addition to respiratory depression and the irregular breathing, other contributing factors may also be involved in the genesis of sudden death. The capacity of opioid-induced respiratory depression to lead to secondary hypoxemia (through hypoventilation and acidosis) has been well-documented [53–55]. For instance, Henderson et al. reported that intravenous injection of fentanyl at 25 μg/kg induced persistent hypoventilation beginning 3–5 minutes post-injection, which was associated with hypoxemia, hypoventilation, and acidosis occurring several minutes later in conscious rats [54]. To our knowledge, the impact of exposure to CRF aerosol on blood gases has not been investigated. However, one study showed that IV injection of CRF (20 μg/kg) markedly depressed ventilation associated with significant changes in the parameters of arterial blood gases including hypoxemia, hypercapnia and acidosis [13]. We can infer that CRF exposure will produce similar blood gases changes, although arterial blood gases measurement was not conducted in this study. In addition to the hypoventilation, opioid-induced increase in pulmonary vascular resistance can decrease alveolar perfusion [54–56], which potentially participate in the induction of hypoxemia and hypercapnia. Actually, opioid overdose could result in brain hypoxemia (along with hypercapnia and acidosis) to promote cardiorespiratory failure and arrest [57–60]. On the other hand, fentanyl has been reported to suppress myocardial contractility in humans [61,62] and directly depress cardiac excitation-contraction coupling at the cellular level in animals *in vitro* [63,64]. In consistent with previous findings [65,66], CRF exposure in this study also induced severe arrhythmia and hypotension prior to the death (Figure 6). Therefore, CRF exposure leads to sudden death in this study presumably via its direct effects on respiratory control and myocardial contractility, as well as its indirect impacts resulting from brain hypoxemia, as mentioned above.

In summary, our results demonstrate that exposure to lethal concentrations of CRF induces three phases of cardiorespiratory responses, ultimately leading to sudden death in anesthetized and spontaneously breathing rats. Respiratory failure is characterized by ataxic breathing prior to the death. Our findings establish a rat model for acute CRF exposure-induced sudden death following bradypnea and ataxic breathing, along with hypotension and bradycardia. These findings broaden our current knowledge about the fatality of CRF exposure and the progression of cardiorespiratory failure leading to sudden death. More importantly, they may advance further mechanistic studies and benefit the development of effective countermeasures.

## Figures and Tables

**Table 1:** The baseline values of cardiorespiratory variables in the rats exposed to Veh, and CRF (mean ± SE).

Variables	Veh	CRF
	(N = 5)	(N = 6)
VE (ml min^−1^ kg^−1^)	847 ± 81	835 ± 44
fR (br min^−1^)	116 ± 7	125 ± 5
VT (ml kg^−1^)	7.3 ± 0.4	6.7 ± 0.3
TI (s)	0.28 ± 0.03	0.23 ± 0.02
TE (s)	0.25 ± 0.04	0.26 ± 0.02
TI/TTot	0.53 ± 0.06	0.47 ± 0.04
MABP (mmHg)	82 ± 5	85 ± 6
HR (bpm)	357 ± 11	379 ± 12

## Data Availability

Data will be made available on request.
